# Characterizing hippocampal dynamics with MEG: A systematic review and evidence‐based guidelines

**DOI:** 10.1002/hbm.24445

**Published:** 2018-10-31

**Authors:** Emily Ruzich, Maité Crespo‐García, Sarang S. Dalal, Justin F. Schneiderman

**Affiliations:** ^1^ Department of Clinical Neurophysiology and MedTech West, Institute of Neuroscience and Physiology Sahlgrenska Academy & the University of Gothenburg Gothenburg Sweden; ^2^ MRC Cognition and Brain Sciences Unit University of Cambridge Cambridge UK; ^3^ Center of Functionally Integrative Neuroscience Aarhus University Aarhus C Denmark

**Keywords:** beamforming, deep sources, deep structures, hippocampus, magnetoencephalography, MEG, neural oscillations, source localization, theta

## Abstract

The hippocampus, a hub of activity for a variety of important cognitive processes, is a target of increasing interest for researchers and clinicians. Magnetoencephalography (MEG) is an attractive technique for imaging spectro‐temporal aspects of function, for example, neural oscillations and network timing, especially in shallow cortical structures. However, the decrease in MEG signal‐to‐noise ratio as a function of source depth implies that the utility of MEG for investigations of deeper brain structures, including the hippocampus, is less clear. To determine whether MEG can be used to detect and localize activity from the hippocampus, we executed a systematic review of the existing literature and found successful detection of oscillatory neural activity originating in the hippocampus with MEG. Prerequisites are the use of established experimental paradigms, adequate coregistration, forward modeling, analysis methods, optimization of signal‐to‐noise ratios, and protocol trial designs that maximize contrast for hippocampal activity while minimizing those from other brain regions. While localizing activity to specific sub‐structures within the hippocampus has not been achieved, we provide recommendations for improving the reliability of such endeavors.

## 1 INTRODUCTION

The hippocampus has been established as an essential brain structure for several types of memory and memory‐related functions, including episodic and autobiographical memory (Burgess, Maguire, & O'Keefe, 2002; Taylor, Donner, & Pang, 2012), as well as working memory, association, recognition, and recent recollection (Axmacher, Elger, & Fell, 2008; Fell, Ludowig, Rosburg, Axmacher, & Elger, 2008; Monk et al., 2002; Mormann et al., 2008; Nyberg & Eriksson, 2015; Scoville & Milner, 1957; Stark & Squire, 2000) while having a central role for the related ability of spatial navigation (Ekstrom et al., 2005; Iglói, Doeller, Berthoz, Rondi‐Reig, & Burgess, 2010; Lövdén et al., 2011). Because the hippocampus processes information from all sensory systems and is involved in various higher‐order brain functions, it acts as a hub and is connected to several neural systems mediating attention, decision‐making, and perception across the senses (Battaglia, Benchenane, Sirota, Pennartz, & Wiener, 2011). As a result of this, the hippocampus is also often implicated in a variety of neurodegenerative diseases such as Alzheimer's disease and dementia and is frequently involved as a generator in conditions such as medial temporal lobe (partial) epilepsy (Sitoh & Tien, 1998; Velez‐Ruiz & Klein, 2012). For these reasons, the hippocampus is an important and complex focus of study across several disciplines.

The measurement of hippocampal activity using magnetoencephalography (MEG), while potentially challenging, has certain advantages, such as the possibility of better describing its spectro‐temporal dynamics. Many researchers are now undertaking this venture with reported, if somewhat mixed, success. In a recent review, Pu, Cheyne, Cornwell, and Johnson (2018) discuss theoretical and experimental outcomes of MEG measurements of hippocampal activity to evaluate the strength of the evidence for MEG sensitivity to signals originating in the hippocampus. They summarize simulation studies suggesting that hippocampal magnetic fields can be detected by MEG sensors at the surface and distinguished from other neocortical and parahippocampal activities when applying source localization methods. They also examined several empirical studies showing hippocampal‐related effects with MEG, though their goal was to compare them with those found with other modalities such as fMRI. In contrast, we follow a different approach in the present review: we scrutinized empirical studies, searching for common experimental and analytical factors that could increase the chances to observe hippocampal modulations. Because the reviewed literature is rather heterogeneous (i.e., including healthy subject and patient groups, experimental and clinical paradigms, analysis and modeling methods that have been attempted to date, and so forth), the criteria for characterizing normal (i.e., nonepileptiform or interictal) hippocampal activations with MEG would remain unclear without a systematic approach. Therefore, we set out to systematically review the state of recent literature relating to MEG in a targeted attempt to highlight specific considerations and pinpoint crucial recommendations for improving the likelihood of success in future studies that aim to investigate hippocampal function. In addition, while other reviews also provide guidelines for MEG (e.g., Gross et al., 2013; Hari et al., 2018; Hari & Salmelin, 2012), ours are specific to the study of the hippocampus and within the context of academic research.

### 1.1 Hippocampal electrophysiological activity

Electrophysiological activity in the hippocampus shows very characteristic oscillatory patterns at several frequencies (Colgin, 2016), although it is often dominated by theta waves. Because hippocampal bodies are located deep within the brain, direct study of these rhythms can be difficult in humans; instead, animal models have laid the groundwork for our understanding of hippocampal roles and activations related to spatial navigation and learning tasks. Notably, theta rhythms appear to play a significant role in these hippocampal functions (Buzsáki, 2002) and are hypothesized to drive many aspects of global oscillatory dynamics (Fries, 2005). While animal models have demonstrated that the theta band (spanning 4–12 Hz in rodents) is a dominant component of hippocampal activity (Vertes, Hoover, & Viana Di Prisco, 2004), evidence suggests that the equivalent activity in humans includes the classic 4–8 Hz band as well as lower frequencies (1–4 Hz) (Jacobs, 2014; Watrous et al., 2013).

Clinicians and researchers have, on the other hand, classically defined a 4–8 Hz theta band for electromagnetic scalp recordings in humans (Dondey & Klass, 1974), and often reported the strongest topographical component (~6 Hz) at frontal midline sites. A review of frontal‐midline theta describes a variety of functional tasks, including memory, navigation, and attention, that can alter spectro‐temporal features of this frequency band, and furthermore reveals that this rhythm can be modified by drugs and certain psychiatric conditions (Mitchell, McNaughton, Flanagan, & Kirk, 2008). Several studies have also found changes in theta power at frontal and other scalp sites during tasks that may engage the hippocampus, demonstrating a link between human theta band and complex maze navigation (Kahana, Sekuler, Caplan, Kirschen, & Madsen, 1999), exploratory behavior (Orekhova, Stroganova, Posikera, & Elam, 2006), working memory tasks (Onton, Delorme, & Makeig, 2005), episodic memory retrieval (Hanslmayr, Staudigl, Aslan, & Bäuml, 2010; Lee & Zhang, 2014), and attention to social and nonsocial inputs (Orekhova et al., 2006). Nevertheless, the relationship between hippocampal function and theta oscillatory activity measured at the scalp is complex: the human theta band may not be exclusively tied to hippocampal activity, but rather to a more extensive network that includes the hippocampus. Indeed, connectivity analyses show that the prefrontal cortex, medial temporal lobe, and some subregions of the parietal cortex are also tied to changes in theta (Lee & Zhang, 2014). In addition, frequencies outside of theta (with lower amplitude) may also be correlates of hippocampal activation in humans. For instance, gamma activity (30–100 Hz) can be linked to hippocampal circuits and may represent the formation of new declarative memory (Axmacher, Mormann, Fernández, Elger, & Fell, 2006), and hippocampal atrophy is associated with changes in high and low alpha power ratios (Moretti et al., 2012). Taken together, while theta‐band oscillations receive the most attention when it comes to electrophysiological signaling from the hippocampus, there is evidence for hippocampal‐related alpha‐ and gamma‐band activations that are of functional relevance as well.

### 1.2 Neuroimaging investigations of hippocampal function

Currently, the role of the human hippocampus is primarily studied with functional MRI (fMRI) and positron emission tomography (PET), but these techniques capture changes in hemodynamics and metabolic function that are not always clearly associated with hippocampal oscillations (Angenstein, Kammerer, & Scheich, 2009; Ekstrom, Suthana, Millett, Fried, & Bookheimer, 2009); their limited temporal resolution furthermore restricts the detail with which hippocampal physiology can be explored. Questions regarding the timing, oscillations, and network signaling involved in such activations therefore remain open.

Some researchers make use of intracranial EEG (iEEG) from neurosurgery patients to more directly measure electrical activity from the human hippocampus. This approach has led to new insights (for review, see Colgin, 2016 and Jacobs, 2014). However, it is only possible to investigate hippocampal activity with iEEG in patients that already have electrode implants for clinical purposes. Therefore, a significant limitation to such research is that iEEG electrode positions offer a limited spatial coverage and are dictated by pathophysiological concerns, and further that any underlying pathophysiology may limit the relevance of findings to neurotypical populations. Further complicating the issue is the fact that electrodes are generally implanted in different areas across patients (and not always in the hippocampus); robust group statistics are therefore limited. From a practical perspective, the recruitment of such a clinical sample is generally lengthier than in experiments with healthy participants (Arnulfo, Hirvonen, Nobili, Palva, & Palva, 2015; Zaveri, Duckrow, & Spencer, 2000).

MEG and electroencephalography (EEG) noninvasively measure the respective magnetic fields and electric potentials generated by neuronal currents. Despite arising from measurements of related electromagnetic physiological sources, one advantage MEG has over EEG is that the magnetic signal undergoes less distortion while passing through tissue boundaries as compared to the electric signal (Hämäläinen, Hari, Ilmoniemi, Knuutila, & Lounasmaa, 1993; Vorwerk et al., 2014). The potential to detect activity with fine temporal resolution makes these methods well‐suited to investigate many transient and oscillatory hippocampal processes of interest. The possibility of localizing these signals and describing hippocampal and parahippocampal network function has emerged with the advent of dense, whole‐head MEG and EEG sensor arrays, along with advanced modeling tools. MEG is particularly promising as an appropriate method for investigating the hippocampus for certain kinds of studies, particularly those focusing on oscillatory and transient activity. The superior spatial resolution of magnetic resonance and blood‐oxygen‐level‐dependent (BOLD) contrast imaging is complemented by the high temporal resolution brought about by the direct measurement of neural signals intrinsic to MEG and EEG. Researchers are now pushing the technology to its limits, making use of advances in hardware as well as analytic software.

### 1.3 The potential of MEG for hippocampal research

**Figure 1:**
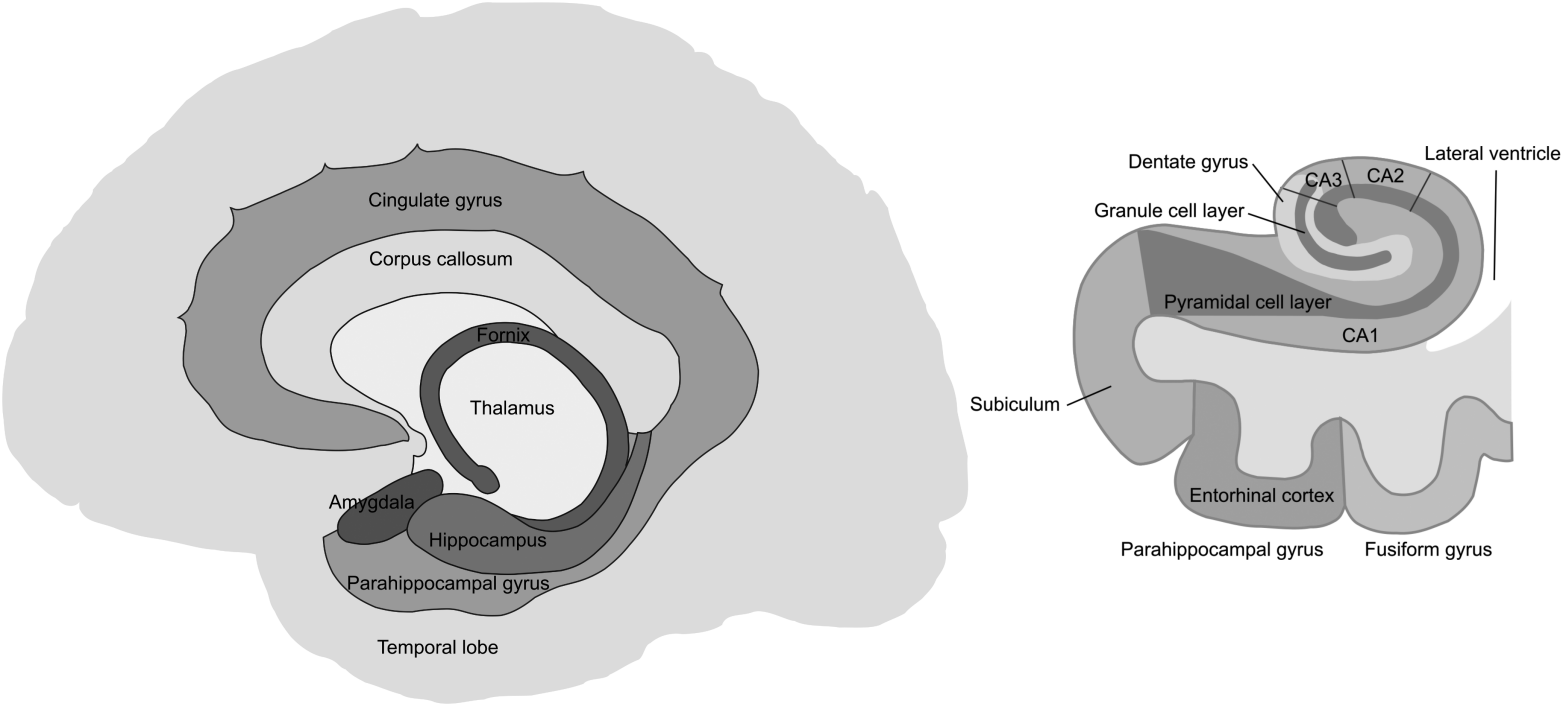
Schematic depictions of the location of the hippocampal formation relative to other brain structures (left panel) and transverse section of the medial temporal lobe with the main hippocampal subfields and parahippocampal cortex in a coronal view (right panel)

The human hippocampal formation is a bilateral structure composed of two elongated curved bodies oriented along the inferotemporal floor of the temporal horns of the lateral ventricles. Each body is positioned just behind the homolateral amygdala and posteriorly extends to the fornix, the main efferent system of the hippocampus (Figure 1, left panel). The principal hippocampal subfields are the *Cornu Ammonis* divisions (CA1, CA2, and CA3), the dentate gyrus, and the subiculum; together, they have the appearance of an S‐shaped scroll in coronal slices of the brain (Figure 1, right panel). The structure is formed by an invagination of the tissue into the medial part of the temporal cortex, rendering it well beneath the cortical surface of the brain. The subiculum connects ventrally with the parahippocampal gyrus, which in its anterior part contains the entorhinal cortex, the major gateway between the hippocampus and the neocortex. With only three cellular layers, the cortical structure of the hippocampus (or *archicortex*) is more primitive than that of the neocortex. Furthermore, the CA subfields and dentate gyrus contain only a single layer of neurons, pyramidal, and granular cells, respectively. However, the main axes of the dendritic trees from the pyramidal cells are arranged in parallel and oriented perpendicularly to the curved surface of the hippocampus (see Meyer et al., 2017 for more details).

MEG is primarily sensitive to the magnetic fields produced by summed postsynaptic electric currents stemming from the architecture of pyramidal neurons. Current MEG technology predominantly utilizes superconducting quantum interference devices (SQUIDs) to sensitively measure changes in these minute fields within a magnetically shielded room. Additional sensitivity to deeper structures, including the hippocampus, can be achieved for instance by relying more heavily on magnetometers, which have a longer reach than planar gradiometers (Quraan, Moses, Hung, Mills, & Taylor, 2011). Converting these magnetic measurements into neural activation maps requires projecting them onto an anatomical model of the brain through a series of calculations utilizing additional information from a structural MRI or a canonical mesh. Each source reconstruction technique involves various assumptions that aim to constrain the final depiction of brain activity. However, reconstructing hippocampal activations from MEG data presumes MEG sensitivity to this deep structure.

In the past, there has been some skepticism regarding the sensitivity of MEG (and EEG) to deep structures (White, Congedo, Ciorciari, & Silberstein, 2012). Some have claimed that such nonsuperficial brain regions cannot be accessed by these methods due to the decay of magnetic and electric field magnitudes as a function of depth, as well as the different arrangement of neurons in contrast to the neocortex (Williamson & Kaufman, 1981). Nevertheless, the hippocampus has a neuronal density 2.5 times higher than that of typical neocortical gray matter (Attal et al., 2007), which may partially counteract the depth attenuation, and modern anatomical head modeling techniques perform more robustly at depth (Wolters et al., 2006).

**Figure 2:**
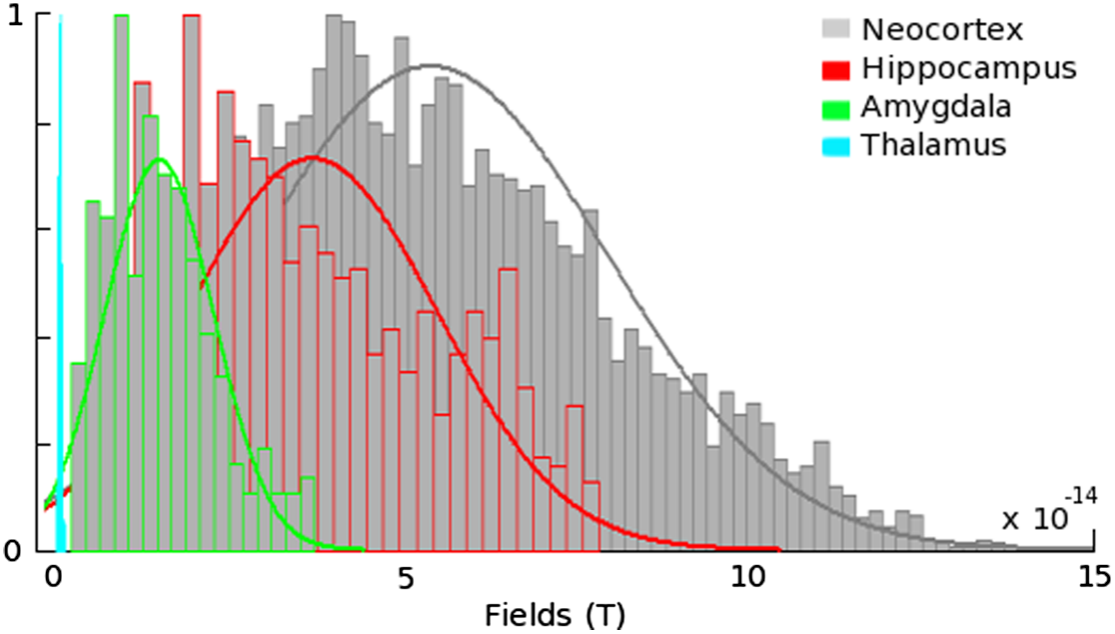
Simulated data of magnetic fields indicate that hippocampal volumes produce a signal that, while lower in magnitude than that of the neocortex, should nonetheless be robust enough for detection with MEG. These simulations take into account a number of variables including anatomical geometry, source‐to‐sensor gain matrix, and current dipole moment density (adapted from “Assessment of subcortical source localization using deep brain activity imaging model with minimum norm operators: A MEG study,” by Attal & Schwartz, 2013, PLoS One, 8, e59856) [Color figure can be viewed at http://wileyonlinelibrary.com]

Despite the limitations of MEG, there is substantive hope that this technology might be used reliably for measuring the temporal dynamics of deeper sources as much as for cortical structures, particularly from the hippocampus. In addition to MEG signals being less spatially distorted than EEG, sophisticated forward modeling of MEG sensor data has led researchers to conclude that contributions from the hippocampus and hippocampal networks should be detectable with MEG (Attal & Schwartz, 2013) (Figure 2).

Indeed, rare recordings of simultaneous MEG and intracranial depth electrodes in epilepsy surgery patients yield strong evidence that hippocampal activity generates measurable MEG signals. Santiuste et al. (2008) demonstrated that individual epileptic spikes originating from the hippocampus can often be observed simultaneously with MEG, though spikes of lower amplitude or smaller spatial extent were less likely to be observed. They speculate that different spatial orientations of different spikes could be another factor influencing spike detection with MEG. Of course, neurotypical activity generally has lower signal amplitude than epileptic spikes. However, Dalal et al. (2013) observed in a different set of simultaneous MEG and depth electrode recordings that spontaneous and apparently neurotypical theta oscillations isolated to hippocampal depth electrodes also appeared in clusters of MEG sensors, exhibiting a bipolar correlation pattern (involving frontal and posterio‐temporal sensors) consistent with a relatively deep brain source. While the correlation was established over a total of 24 min of recording, the relationship could be clearly seen in continuous recordings. Taken together, these reports provide evidence that both pathological and neurotypical hippocampal activity can indeed be observed with MEG, sometimes even in raw data. While this provides an empirical foundation for detecting hippocampal activity with MEG, it emphasizes that the challenge lies in confidently localizing hippocampal activity from MEG data alone, that is, without the benefit of confirmatory evidence from intracranial recordings.

### 1.4 Key anatomical, methodological, and analytical challenges

Limitations of using MEG to investigate deep signals include low signal‐to‐noise ratios (SNRs), co‐registration issues, and the presence of artifacts (e.g., environmental sources, heartbeat, eye movements, and blinks) producing magnetic signals of equal or larger magnitude than the neural sources (Gross et al., 2013). These issues can be particularly challenging for reconstructing complex, transient, or deep‐source activity. Beyond this, MEG, and in fact nearly all neuroimaging methods, rely on response averaging to improve SNRs, which limits the ability of these methods to accurately observe transient phenomena — such as, for instance, the mechanisms associated with learning, which may not yield precisely event‐related responses and are likely to vary greatly across individuals.

The challenges of accurately localizing activity to the hippocampus are indeed greater than those common to localizing activity to cortical structures. In addition to the previously discussed general limitations of MEG imaging and source localization techniques, the quasi‐cylindrical geometry of the hippocampal anatomy is such that electromagnetic signals may cancel each other out if opposing subfields are simultaneously activated (Williamson & Kaufman, 1981). The structure's distance from magnetic sensors and position beneath key temporal regions that are regularly activated during sensory and linguistic tasks mean that hippocampal signals can be comparatively faint and difficult to isolate. Furthermore, the spherical head models historically used for MEG/EEG source localization are particularly prone to error at depth (Fuchs, Drenckhahn, Wischmann, & Wagner, 1998). However, the density and orientation of pyramidal neurons in the hippocampus (Duvernoy, Cattin, & Risold, 2013) make it a good candidate for detection with MEG. In fact, simulated data with physiologically constrained models indicate hippocampal sources should generate MEG signal magnitudes that are only marginally weaker than that which is consistently detected from the neocortex (Attal & Schwartz, 2013; Figure 2).

Beyond technical and physical caveats, several additional limitations relating to analysis and localization methods have the potential to prove particularly troublesome for detecting hippocampal activations with MEG. For instance, many early clinical studies used equivalent dipole methods to localize epileptiform spike activity to a seizure focus, and initially, these methods were also used in general research studies as well. However, while suitable for modeling early evoked fields located near the cortical surface, localization methods using a simple dipole are now considered to be an oversimplification, whereas minimum‐norm and beamforming‐based inverse methods are more appropriate for components that are likely to be from deeper or less dominant sources, have longer, later, or more variable duration, are generated by nonfocal distributed sources, or simply are largely uncharacterized and unknown based on current understanding (Chatani et al., 2016; Gramfort et al., 2014; Henson, Mattout, Phillips, & Friston, 2009; Lalancette, Quraan, & Cheyne, 2011; Pellegrino et al., 2018).

### 1.5 Current understanding

A number of reviews touch on the use of MEG to record activity from human hippocampal sources. For example, several on epilepsy (Sitoh & Tien, 1998; Velez‐Ruiz & Klein, 2012) and memory and cognition (Taylor et al., 2012) place hippocampal activity as measured by MEG in a broader context. Perhaps the most definitive work on the feasibility of detecting MEG signals from deep structures has been done by Attal and colleagues, whose papers make efforts to evaluate how the orientation, location, and architecture of deep brain structures, including the hippocampus, can impact the localization of signals from these areas. They argue that isolating deep brain activity is a challenging but possible undertaking and that it is heavily reliant on methodological considerations, including improved forward structural models and inverse solutions grounded in realistic biophysical neural models. This work, in addition to the group's investigations of different inverse operators on simulation data (Attal et al., 2007; Attal & Schwartz, 2013), demonstrates the plausibility of such an undertaking. However, a comprehensive investigation of the current state of MEG used to study hippocampal activity is currently lacking.

### 1.6 Aims

The current review examines the existing literature to systematically collect and critically analyze experimental studies using MEG to resolve hippocampal activity. Our main objective was to determine how state‐of‐the‐art MEG has been used to quantify normal (i.e., nonepileptiform or interictal) activity from alleged hippocampal sources to describe current developments and challenges in the field, as well as potential future uses of this technology. We furthermore aimed to extract different methodological aspects that are common across studies claiming detection of hippocampal sources and explore the strength of those claims to finally identify the requirements and best conditions for which hippocampal activity can be reliably detected and localized with MEG. A final goal was to guide future study design and provide feasibility assessments with recommendations gleaned from our approach.

## 2 METHODS

We conducted a search of PubMed within all indexed fields for the terms “magnetoencephalography”, “MEG” AND “hippocamp*” (to include hippocampal, hippocampus, and so forth). Prior to independent evaluation of the returned results, a list of criteria for inclusion and exclusion was developed. An initial screening removed any (a) duplicate hits, (b) nonhuman research or research that did not use real data from human participants, and (c) non‐English language manuscripts. Further screening omitted (d) book chapters, conference proceedings, and other nonpeer‐reviewed publications; (e) letters, commentary, reviews, and other papers, that is, those not including original novel research; (f) studies that included participants under the age of 18 or over the age of 65, as these groups may have different physiological responses and/or neuroanatomy (Driscoll et al., 2003; Gómez & Edgin, 2016); and (g) studies with a focus on epileptiform activity, as these signals can be several orders of magnitude larger than the typical signals from hippocampus (Knowlton et al., 1997). Note that other clinical studies, including studies of other patient groups and interictal studies of patients with epilepsy, were included. Following this, the remaining manuscripts were tested for eligibility: (h) studies were included if they had significant a priori focus on the hippocampus or medial temporal lobe (MTL), judged by evaluating the title, abstract, and introduction; (i) if there was significant use of MEG technology, and if the MEG system used was a modern, low‐Tc SQUID sensor‐based, whole‐head system. Finally, studies were excluded if: (j) there was no source localization method, or (k) dipole fitting was implemented for source reconstruction.

The literature search and quality assessment were both independently performed, and the final set of articles found were subsequently compared. In cases of disagreement, all four co‐authors were consulted to reach a final decision. Following this resolution process, data were extracted from each included paper and tabulated; study characteristics and quality were considered at this stage. Characteristics included information about the MEG system utilized, the task or resting state paradigm employed, the participant groups studied (including the presence of any clinical diagnosis), the analysis methods implemented, and any additional confirmatory measures or methods used by the researchers as part of the study. This data was then qualitatively synthesized so that resultant findings could be interpreted and potential sources of bias could be explored.

## 3 RESULTS

**Figure 3:**
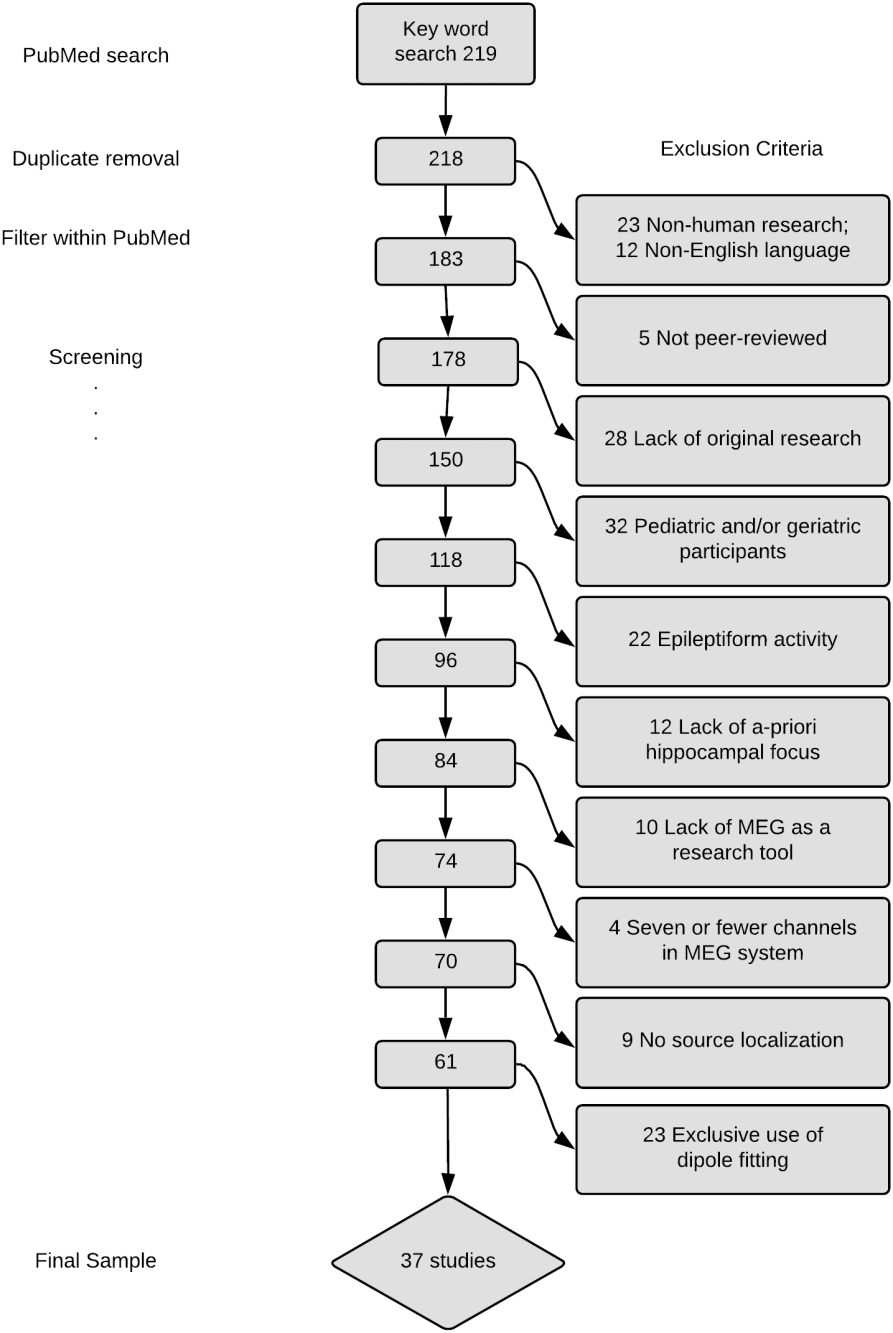
Selection process for review: Studies were screened to determine that basic requirements were met and then selected based on an independent quality assessment that used prespecified inclusion and exclusion criteria

The results of our search and review process are documented in Figure 3. The final number of studies included for review was 37. Table 1 includes descriptive details of the studies.

**Table 1:** List of studies that met criteria for inclusion: Listed chronologically, with details about the number and type of participants, the MEG system used, and the source localization method

Authors	Title	Year	Subjects	MEG system	Localization method	Study design
Filbey, Holroyd, Carver, Sunderland, Cohen	A magnetoencephalography spatiotemporal analysis of neural activities during feature binding.	2005	16 NT	275 first‐order axial grads CTF Omega	SAM beamformer, multisphere head model^a^	Feature‐binding memory task Measured changes in 3.5–7 Hz power
Guderian, Düzel	Induced theta oscillations mediate large‐scale synchrony with mediotemporal areas during recollection in humans.	2005	9 NT	148 mags 4D Neuroimaging Magnes 2,500 WH	Forward triangle models based on cortical surface of MNI brain template, linear least squares minimum norm	Memory paradigm with faces evaluating source retrieval Measured across‐trials phase‐consistent 3–8 Hz oscillatory activity
Martin, McDaniel, Guynn, Houck, Woodruff, Bish, Moses, Kicić, Tesche	Brain regions and their dynamics in prospective memory retrieval: a MEG study.	2007	5 NT	306 channel Elekta (204 planar grads, 102 mags)	Forward model based on MNI brain template, L1‐norm minimum current estimate procedure (also used ECD)	Tasks evaluating prospective and retrospective memory relative to recognition of oddball stimuli Measured alpha and theta band activity
Cornwell, Johnson, Holroyd, Carver, Grillon	Human hippocampal and parahippocampal theta during goal‐directed spatial navigation predicts performance on a virtual Morris water maze.	2008	15 NT	275 first‐order axial grads CTF Omega	SAM beamformer, multisphere head model, normalization of active windows with pretrial baseline windows	Virtual Morris water maze Measured 4–8 Hz oscillations during goal‐directed navigation and aimless movements
Moses, Ryan, Bardouille, Kovacevic, Hanlon, McIntosh	Semantic information alters neural activation during transverse patterning performance.	2009	18 NT	151 first‐order grads CTF Omega	SAM beamformer, multisphere head model^a^	Transverse patterning task Used event‐related beamforming combined with partial least squares approach
Riggs, Moses, Bardouille, Herdman, Ross, Ryan	A complementary analytic approach to examining medial temporal lobe sources using magnetoencephalography.	2009	13 NT	151 first‐order grads CTF Omega	SAM beamformer, multisphere head model^a^	Recognition of old and new complex scenes Measured 4–8 Hz event‐related responses, inter‐trial coherence, and event‐related and nonevent‐related spectral power
Cornwell, Salvadore, Colon‐Rosario, Latov, Holroyd, Carver, Coppola, Manji, Zarate, Grillon	Abnormal hippocampal functioning and impaired spatial navigation in depressed individuals: Evidence from whole‐head magnetoencephalography.	2010	19 patients with depression; 19 NT	275 first‐order axial grads CTF Omega	SAM beamformer, multisphere head model	Virtual Morris water maze Measured 4–8 Hz oscillatory activity
Fujioka, Zendel, Ross	Endogenous neuromagnetic activity for mental hierarchy of timing.	2010	13 healthy individuals with musical expertise	151 first‐order grads CTF omega	Multisphere forward model based on template brain MRI, SAM beamformer	Auditory timing detection task Measured averaged evoked magnetic fields
Hanlon, Houck, Pyeatt, Lundy, Euler, Weisend, Thoma, Bustillo, Miller, Tesche	Bilateral hippocampal dysfunction in schizophrenia.	2011	13 patients with schizophrenia; 13 NT	275 first‐order axial grads CTF Omega	sLORETA, Curry v6 BEM model (3‐shell?) with forward model consisting of a 3D grid of fixed dipoles, with points spaced 5 mm apart	Transverse patterning task with elemental contrast Measured left‐ and right‐source strength and latency
Kveraga, Ghuman, Kassam, Aminoff, Hämäläinen, Chaumon, Bar	Early onset of neural synchronization in the contextual associations network.	2011	9 NT	306 channel Elekta (204 planar grads, 102 mags)	MNE, single‐layer BEM, fMRI‐constrained anatomical ROIs (including PHC)	Contextual processing task Measured phase synchrony
Poch, Fuentemilla, Barnes, Düzel	Hippocampal theta‐phase modulation of replay correlates with configural‐relational short‐term memory performance.	2011	8 NT	275 first‐order axial grads CTF Omega	Single‐shell forward model based on MNI brain template, LCMV beamformer	Delay match‐to‐sample recall task Measured phase coupling of 5–7 Hz oscillatory activity
Quraan, Moses, Hung, Mills, Taylor	Detection and localization of hippocampal activity using beamformers with MEG: a detailed investigation using simulations and empirical data.	2011	15 NT	151 first‐order grads CTF Omega	Scalar and vector beamformers, multisphere head model, with subtraction of control from experimental image	N‐back task Measured transient evoked signals
Cornwell, Arkin, Overstreet, Carver, Grillon	Distinct contributions of human hippocampal theta to spatial cognition and anxiety.	2012	25 NT	275 first‐order axial grads CTF Omega	SAM beamformer, multisphere head model, experimental relative to baseline image ratio, hippocampal ROI	Water maze combined with aversive feedback Measured modulation of 4–8 Hz and 2–6 Hz oscillations
Kaplan, Doeller, Barnes, Litvak, Düzel, Bandettini, Burgess	Movement‐related theta rhythm in humans: Coordinating self‐directed hippocampal learning.	2012	17 NT	275 first‐order axial grads CTF Omega	Single‐shell forward model based on template brain MRI, LCMV beamformer	Virtual arena spatial navigation task. Measured modulation of 4–8 Hz oscillatory activity
Luckhoo, Hale, Stokes, Nobre, Morris, Brookes, Woolrich	Inferring task‐related networks using independent component analysis in magnetoencephalography.	2012	12 NT	275 first‐order axial grads CTF Omega	LCMV beamformer, single‐shell BEM, Hilbert transform and temporal independent component analysis	N‐back task, resting state Measured functional connectivity in 4–8 Hz band
Mills, Lalancette, Moses, Taylor, Quraan	Techniques for detection and localization of weak hippocampal and medial frontal sources using beamformers in MEG.	2012	22 NT	151 first‐order grads CTF Omega	Minimum variance vector beamformer, multisphere head model, with subtraction of image and sensor control data from experimental data	N‐back task, transverse patterning task Measured evoked signals
Van Lutterveld, Hillebrand, Diederen, Daalman, Kahn, Stam, Sommer	Oscillatory cortical network involved in auditory verbal hallucinations in schizophrenia.	2012	12 patients with schizophrenia	151 first‐order axial grads CTF Omega	SAM beamformer, multisphere head model^a^	Resting state—Eyes closed, with spontaneous auditory verbal hallucinations Measured modulation of 0.5–4, 4–8, 8–13, and 13–30 Hz power bands
Guitart‐Masip, Barnes, Horner, Bauer, Dolan, Düzel	Synchronization of medial temporal lobe and prefrontal rhythms in human decision making.	2013	20 NT	275 first‐order axial grads CTF Omega	Single‐shell forward model based on template brain MRI, LCMV beamformer	Decision‐making task Measured modulation of 4–8 Hz oscillatory activity
Hung, Smith, Taylor	Functional dissociations in prefrontal–hippocampal working memory systems.	2013	20 NT	151 first‐order axial grads CTF Omega	Minimum‐variance beamformer, multisphere head model, pseudo‐Z, contrast with baseline	N‐back task
Olsen, Rondina, Riggs, Meltzer, Ryan	Hippocampal and neocortical oscillatory contributions to visuospatial binding and comparison.	2013	24 NT	151 first‐order axial grads CTF Omega	SAM beamformer, multisphere head model^a^, AAL grid equivalent across participants	Encoding and retrieval paradigm Measured 2–7 Hz oscillatory activity
Cornwell, Overstreet, Grillon	Spontaneous fast gamma activity in the septal hippocampal region correlates with spatial learning in humans.	2014	24 NT	275 first‐order axial grads CTF Omega	SAM beamformer, multisphere head model	Virtual Morris water maze, resting state Measured 80–140 Hz high‐gamma ripple modulation
Dunkley, Doesburg, Sedge, Grodecki, Shek, Pang, Taylor	Resting‐state hippocampal connectivity correlates with symptom severity in posttraumatic stress disorder.	2014	23 patients with PTSD; 21 healthy military servicemen	151 first‐order grads CTF Omega	Vector beamformer, multisphere head model, AAL grid equivalent across participants	Resting state (pre and posttriggering stimuli session) Measured functional connectivity in 80–150 Hz band (along with other frequency bands)
Huang, Yurgil, Robb, Angeles, Diwakar, Risbrough, Nichols, McLay, Theilmann, Song, Huang, Lee, Baker	Voxel‐wise resting‐state MEG source magnitude imaging study reveals neurocircuitry abnormality in active‐duty service members and veterans with PTSD.	2014	25 patients with PTSD; 12 healthy military servicemen; 18 healthy civilians	306 channel Elekta (204 planar grads, 102 mags)	High‐resolution Fast‐VESTAL MEG source imaging with single‐shell BEM mesh	Resting state Measured power in high‐ and low‐frequency oscillatory bands
Kaplan, Bush, Bonnefond, Bandettini, Barnes, Doeller, Burgess	Medial prefrontal theta phase coupling during spatial memory retrieval.	2014	17 NT	275 first‐order axial grads CTF Omega	Single‐shell forward model based on template brain MRI, LCMV beamformer	Virtual arena spatial navigation task Measured modulation of inter‐regional 4–8 Hz phase coupling
Lozano‐Soldevilla, ter Huurne, Cools, Jensen	GABAergic modulation of visual gamma and alpha oscillations and its consequences for working memory performance.	2014	25 NT	275 first order axial grads CTF Omega	DICS beamformer, single‐shell	Working memory task, combined with lorazepam (GABA‐ergic upregulator) Measured modulation of 8–14 and 30–100 Hz oscillations
Cousijn, Tunbridge, Rolinski, Wallis, Colclough, Woolrich, Nobre, Harrison	Modulation of hippocampal theta and hippocampal‐prefrontal cortex function by a schizophrenia risk gene.	2015	25 healthy individuals with elevated genetic risk for schizophrenia; 25 NT	306 channel Elekta (204 planar grads, 102 mags)	LCMV beamformer, multisphere head model, Hilbert transform and temporal independent component analysis	Resting state Measured modulation of intrahippocampal networks oscillatory activity at 4–8 Hz
Garrido, Barnes, Kumaran, Maguire, Dolan	Ventromedial prefrontal cortex drives hippocampal theta oscillations induced by mismatch computations.	2015	17 NT	275 first‐order axial grads CTF Omega	Single‐shell forward model based on template brain MRI, LCMV beamformer	Match‐mismatch task Measured modulation of 4–8 Hz oscillatory activity
Nugent, Robinson, Coppola, Furey, Zarate	Group differences in MEG‐ICA derived resting state networks: Application to major depressive disorder.	2015	49 patients with depression; 25 NT	275 first‐order axial grads CTF Omega	SAM beamformer, multisphere head model	Resting state—eyes closed Measured resting state networks for 14–30 Hz power
Backus, Schoffelen, Szebényi, Hanslmayr, Doeller	Hippocampal‐prefrontal theta oscillations support memory integration.	2016	20 NT	275 first‐order axial grads CTF Omega	DICS beamformer, single‐shell, hippocampal ROI, leadfield reduced by singular value decomposition, MNI grid equivalent across participants	Associative interference memory task Measured 3–7 Hz oscillations
Barascud, Pearce, Griffiths, Friston, Chait	Brain responses in humans reveal ideal observer‐like sensitivity to complex acoustic patterns.	2016	16 NT	275 first‐order axial grads CTF Omega	Minimum norm prior model, DSS component analysis applied to sensor data to maximize the difference between conditions	Auditory pattern detection task Measured responses during regularly‐repeating patterns and random sequences
Chatani, Hagiwara, Hironaga, Ogata, Shigeto, Morioka, Sakata, Hashiguchi, Murakami, Uehara, Kira, Tobimatsu	Neuromagnetic evidence for hippocampal modulation of auditory processing.	2016	17 temporal lobe epilepsy patients; 9 patient controls; 17 NT	306 channel Elekta (204 planar grads, 102 mags)	LCMV beamformer (also used ECD), single‐shell BEM	Passive auditory task Measured auditory‐evoked fields M100 and M400
Crespo‐García, Zeiller, Leupold, Kreiselmeyer, Rampp, Hamer, Dalal	Slow‐theta power decreases during item‐place encoding predict spatial accuracy of subsequent context recall.	2016	12 patients with epilepsy; 13 NT	148 mags 4D Neuroimaging Magnes 2,500 WH	LCMV beamformer, four‐shell BEM, with subtraction of baseline from experimental image, MNI grid equivalent across participants	Spatial navigation task Measured 2–5 Hz power modulations
Mišić, Dunkley, Sedge, Da Costa, Fatima, Berman, Doesburg, McIntosh, Grodecki, Jetly, Pang, Taylor	Posttraumatic stress constrains the dynamic repertoire of neural activity.	2016	23 patients with PTSD; 21 healthy military servicemen; 20 mTBI civilians; 21 healthy civilians	151 first‐order axial grads CTF Omega	Vector beamformer, multisphere head model, AAL grid equivalent across participants	Resting state (pre and posttriggering stimuli session) Measured functional connectivity and local signal variability, focusing on 80–150 Hz oscillations
Badura‐Brack, Heinrichs‐Graham, McDermott, Becker, Ryan, Khanna, Wilson	Resting‐state neurophysiological abnormalities in posttraumatic stress disorder: A magnetoencephalography study.	2017	31 combat veterans with PTSD; 20 healthy combat veterans	306 channel Elekta (204 planar grads, 102 mags)	Bayesian multiple sparse priors approach for source reconstruction	Resting state – Eyes closed Measured 1–54 Hz activity
Kaplan, Bush, Bisby, Horner, Meyer, Burgess	Medial prefrontal‐medial temporal theta phase coupling in dynamic spatial imagery.	2017	16 NT	274 axial grads CTF Omega	Single‐shell forward model based on template brain MRI, LCMV beamformer	Spatial working memory task Measured modulation of inter‐regional 4–7 Hz phase coupling
Khemka, Barnes, Dolan, Bach	Dissecting the function of hippocampal oscillations in a human anxiety model.	2017	20 NT	275 first‐order axial grads CTF Omega	Single‐shell forward model based on template brain MRI, LCMV beamformer, broad‐band (1–150 Hz) spatial filters	Anxiety‐inducing approach‐avoidance conflict task Measured modulation of 1–8 Hz oscillatory activity within a‐priori ROI of bilateral hippocampus
Shah‐Basak, Urbain, Wong, da Costa, Pang, Dunkley, Taylor	Concussion alters the functional processes of visual attention and working memory.	2018	18 patients with concussion; 19 NT	151 first‐order axial grads CTF Omega	Single‐shell forward models based on individual brain MRIs, LCMV beamformer, MNI grid equivalent across participants	N‐back task Measured 1–50 Hz averaged evoked magnetic fields

AAL = automated anatomical labeling; BEM = boundary element model; DICS = dynamic imaging of coherent sources, a beamforming technique for spatial filtering in the frequency domain; DSS = denoising source separation method; grads = gradiometers; ICA = independent component analysis; LCMV = linearly constrained minimum variance, a beamforming technique for spatial filtering; mags = magnetometers; MNE = minimum‐norm estimate; MNI = Montreal neurological institute; MRI = magnetic resonance image; NT = neurotypical; PHC = parahippocampal cortex; PTSD = posttraumatic stress disorder; ROI = region of interest; SAM = synthetic aperture magnetometry, a minimum‐variance adaptive beamformer algorithm; mTBI = mild traumatic brain injury.

a We assumed that papers applying SAM with the CTF software used the implemented multisphere head model.

### 3.1 Search results

Table 1 shows the studies included in this review and broadly defines the MEG system and experiment setup as well as the participant groups utilized. For all included studies in this review, the mean number of subjects per group was 20.6 (*SD* 9.02).

### 3.2 Basic characteristics of included studies

The first noted trend in the data was the uptick of novel studies that met criteria, starting around 2011. From 2005 to 2010, there were eight publications meeting criteria (averaging 1.6 per year); this number increased from 2011 through 2018 to 29 (averaging 3.9 per year, counting 2018 as a half‐year). For the final set of studies, we found that the most common MEG system used was the CTF Omega 275, a system that uses 275 first‐order axial gradiometers. Its forerunner with 151 axial gradiometers, the CTF Omega 151 was also commonly used. Systems implementing combined magnetometers and planar gradiometers, such as the Elekta Neuromag, as well as magnetometer‐only systems, were less commonly used.

From the included studies (keeping in mind that dipole methods were deemed as an exclusion factor), we found that various beamforming methods were by far the most common method of source localization. In particular, minimum variance beamforming methods such as linearly constrained minimum variance (LCMV) and synthetic aperture magnetometry (SAM) were frequently applied. Other source localization methods, such as minimum norm models, were only infrequently used.

We were interested to discover whether there were any key differences between early studies and those published more recently. As noted, most of the studies included in this review were published in the last decade. In fact, we ultimately excluded the majority of early MEG studies because of limitations in technology, source reconstruction methodology, and research focus (many of these early studies concern the localization of spike activity in clinical epilepsy cases). We found only six studies published prior to 2010 that met inclusion criteria (Cornwell, Johnson, Holroyd, Carver, & Grillon, 2008; Filbey, Holroyd, Carver, & Cohen, 2005; Guderian & Düzel, 2005; Martin et al., 2007; Moses et al., 2009; Riggs et al., 2009). These studies all employed simple cognitive paradigms testing normal performance in neurotypical individuals; in the majority of cases, a SAM beamformer was used to localize activity to hippocampal regions.

When we set out to perform this review, we were hopeful that many studies would include additional confirmatory measures that would further support pinpointing hippocampal activity with MEG, for instance with replication via fMRI or combined simultaneous iEEG and MEG. However, we found very few studies that explicitly sought to justify the localization of observed electromagnetic activity to hippocampal sources with additional complementary measures. In fact, only four studies used fMRI in addition to MEG (Barascud, Pearce, Griffiths, Friston, & Chait, 2016; Cousijn et al., 2015; Kaplan et al., 2012; Kveraga et al., 2011). Kveraga et al. (2011) used the same stimuli for both MEG and fMRI, and used the observed fMRI activations for selecting MEG regions of interest for a task aimed at defining the temporal dynamics of visual contextual processing; however, the authors only investigated ROIs from the contextual association network, focusing predominantly on parahippocampal, rather than hippocampal, structures. Similarly, the study on individuals with high risk for schizophrenia described in Cousijn et al. (2015) used a dual‐regression approach and ICA decompositions for both MEG and fMRI data to determine whether resting state parahippocampal networks were comparable between the two methodologies. This study reported a strong negative correlation between theta localized to hippocampal networks (via MEG) and coactivation of the superior frontal gyrus and hippocampal network (via fMRI). Furthermore, participants with the schizophrenia risk gene showed increased hippocampal‐prefrontal connectivity and decreased hippocampal theta. Cousijn et al. (2015) propose that these results might reflect a shift in the pattern of network connectivity, in which increases in hippocampal–prefrontal coupling might be accompanied by less engagement of hippocampal theta with the rest of the hippocampal network, expressed as a local desynchronization. Indeed, hippocampal theta is hypothesized to coordinate hippocampal–prefrontal interactions (Colgin, 2011). Another study employing a virtual navigation paradigm (Kaplan et al., 2012) also found a good agreement between hippocampal fMRI activations during self‐directed memory encoding and power changes in movement‐related theta oscillations localized to the right hippocampus. Periods of the task showing movement‐related theta increases showed increased BOLD signal in the hippocampus, and both measures correlated with the participant's subsequent memory performance. Conversely, Barascud et al. (2016) showed in a study of the temporal dynamics and BOLD response to an auditory pattern recognition task that, while on the whole, the observed activated brain networks were similar when using the two methods, MEG findings included hippocampal signaling, while fMRI results did not. They speculate that this could be due to the limitations of the two methods: perhaps hippocampal activity varies at a timescale that is not detectable by fMRI.

After applying our review criteria, the search retrieved only one paper including data from both iEEG and MEG recordings obtained from patients and healthy participants during the performance of the same spatial memory and navigation task (Crespo‐García et al., 2016). The study also included a simultaneous recording from a single patient who had depth electrodes contacting lateral and mesial parietal, temporal and frontal lobe structures of the left hemisphere. With this combined data set, it was possible to validate MEG power and phase‐connectivity modulations relative to hippocampal sources by performing analogous analysis on iEEG signals.

While fMRI and simultaneous iEEG may seem to be the most commonly used methods of confirming localization findings from MEG, other methodological factors can be addressed that give added confidence to the localization of MEG‐recorded signals to activity in the hippocampus. A number of studies used tasks that originated from classic experiments with animal models that demonstrate hippocampal activity, including the Morris water maze (Cornwell et al., 2010; Cornwell, Arkin, Overstreet, Carver, & Grillon, 2012; Cornwell, Johnson, et al., 2008; Cornwell, Overstreet, & Grillon, 2014; Kaplan et al., 2012; Kaplan et al., 2014), other spatial navigation tasks (Crespo‐García et al., 2016), and a number of working memory tasks (Backus, Schoffelen, Szebényi, Hanslmayr, & Doeller, 2016; Filbey et al., 2005; Hung, Smith, & Taylor, 2013; Kaplan et al., 2017; Lozano‐Soldevilla, Ter Huurne, Cools, & Jensen, 2014; Poch, Fuentemilla, Barnes, & Duzel, 2011; Shah‐Basak et al., 2017). Other studies included in this review used contrast tasks or baseline conditions to subtract activity from irrelevant cortical areas, for instance, in the case of the elemental task found as part of the transverse patterning task (Mills, Lalancette, Moses, Taylor, & Quraan, 2012; Moses et al., 2009). Solving the transverse patterning task demands that participants learn overlapping relations among elements sequentially presented in pairs. Participants should choose between the elements of a pair, but A is only correct when paired with B, B is only correct when paired with C, and C is only correct when paired with A. On the other hand, in the elemental task a sequence of paired elements is also presented but there is no overlapping of elements across pairs. Both tasks are perceptually equivalent and will evoke similar visual responses that can be subtracted. However, only the transverse patterning task is hippocampal dependent because it can be solved by configurational learning when the last pair of elements (C and A) is presented, whereas the elemental task can be solved by learning about the individual elements and may not engage the hippocampus to the same extent. Furthermore, many studies made an effort to desynchronize activity from extra‐hippocampal cortical areas, for example, using a task designed to avoid the presentation of strong visual or auditory stimuli at the time points during which it was hypothesized that hippocampal activity will occur (Chatani et al., 2016; Mills et al., 2012; Poch et al., 2011). Finally, added confidence can come from simulation data, for example, as in the study by Quraan et al. (2011).

Because of the location of the hippocampus and the likelihood of other brain regions swamping activity from this deeper source, we also noted any mention of SNR calculations. This aspect is particularly relevant when applying beamformers because these adaptive spatial filters cannot fully cancel out activities outside the location of interest; therefore, a weak hippocampal source may be masked by activity “leaking” from other stronger sources (e.g., visual cortex). Few modern MEG studies discuss SNR, and in fact, only one study from this review quantified and reported SNR as a part of their simulation results (Mills et al., 2012). In their paper, Mills et al. (2012) designed sinusoidal signals with different amplitudes at known spatial locations and deliberately added them at concrete latencies on visual evoked fields (i.e., noise). Furthermore, because they knew the real location of the simulated signals, they could investigate the impact of SNR and leakage on localization accuracy. Of course, in real‐data studies, it is not possible to determine the exact amplitude values of sources and their background activities, but SNR could be approximately inferred. Nevertheless, three other manuscripts also mentioned the importance of this factor in their methodology or discussion (Guderian & Düzel, 2005; Hanlon et al., 2011; Riggs et al., 2009).

Finally, we wanted to understand what types of participants, as well as which clinical populations, were most often the subjects of these studies, as we were interested in what types of research questions involved MEG paradigms to probe hippocampal activity. In addition to healthy neurotypical volunteers, patient populations included those with epilepsy, schizophrenia, posttraumatic stress disorder (PTSD), and depression. We explore the types of research questions and paradigms used in greater detail in the following sections.

### 3.3 Neurotypical studies: A detailed evaluation

The first group of 25 studies uncovered by this review consisted of those that had research questions about hippocampal activity in healthy neurotypical participants. These can be further divided into studies that focused on methodological considerations; those that examined memory consolidation and retrieval, as well as working memory; and those that investigated perception.

#### 3.3.1 Methodological investigations

In their methodological study, Quraan et al. (2011) used realistic simulations of evoked activity corroborated with real empirical data from an *n*‐back task to conclude that hippocampal activations can, in fact, be detected and accurately localized using a vector beamformer spatial filter (i.e., event‐related minimum variance beamforming methods) and a multisphere head model. This study considered a variety of factors that may have an impact on the success of localization and demonstrate that, in addition to the strength of the neural signal, a number of methodological tweaks can be performed by the researcher to improve accuracy. First, as many studies suggest, increasing the number of trials and group size has a substantial effect— Quraan et al. (2011) recommend at least 150 trials per condition and at least 12 participants per group (refer to Table 1 for reported group sizes of the included studies). They also confirm the importance of designing appropriate contrasts to optimize differences in hippocampal activation over the relatively strong responses of, for example, sensory areas. To further reduce the influence of background brain noise, they advocate the use of adaptive spatial filters, which by definition reduce activity from surrounding areas outside of the region of interest. A follow‐up study (Mills et al., 2012) focused on how to reduce leakage from these strong sources, comparing several contrast subtraction methods. They again used empirical data from an *n*‐back task with a control condition, but also added a transverse patterning task with an elemental control task.

#### 3.3.2 Memory studies

Because previous literature review of both neuroimaging and invasive electrophysiological data has summarized consistent evidence that hippocampus is essential for relational organization and flexible expression of spatial and nonspatial memories (Eichenbaum, 2017), it is not surprising that our methods returned a majority of studies employing a cognitive task relying on mnemonic operations. This proportion is even higher when excluding those focused on clinical populations. The resulting studies covered several aspects of long‐term memory (Backus et al., 2016; Guderian & Düzel, 2005; Kveraga et al., 2011; Martin et al., 2007; Moses et al., 2009; Riggs et al., 2009), spatial navigation (Cornwell et al., 2012; Cornwell et al., 2014; Cornwell, Johnson, et al., 2008; Crespo‐García et al., 2016; Kaplan et al., 2012; Kaplan et al., 2014), and working memory (Barascud et al., 2016; Filbey et al., 2005; Hung et al., 2013; Kaplan et al., 2017; Lozano‐Soldevilla et al., 2014; Luckhoo et al., 2012; Mills et al., 2012; Olsen, Rondina, Riggs, Meltzer, & Ryan, 2013; Poch et al., 2011; Quraan et al., 2011; Shah‐Basak et al., 2017). As might be expected, hippocampal activation correlated with the formation of new relations between visual stimuli (Olsen et al., 2013) and was higher than activation during the processing of nonassociated stimuli or those already linked by semantic relationships (Backus et al., 2016; Mills et al., 2012; Moses et al., 2009). In line with this idea, results from another study suggest that the hippocampus also contributes to the integration of complex temporal auditory patterns, showing increased neural responses for regularly repeated, relative to random, sound sequences in the hippocampus (Barascud et al., 2016).

Within the neurotypical studies, of the 25 papers that claimed success in detecting hippocampal sources, seven used experimental designs involving recognition tasks (Garrido, Barnes, Kumaran, Maguire, & Dolan, 2015; Guderian & Düzel, 2005; Hung et al., 2013; Luckhoo et al., 2012; Martin et al., 2007; Mills et al., 2012; Riggs et al., 2009). Several of these employed classic *n*‐back paradigms (although other methods, such as the transverse patterning task, were also used). MEG activity recorded during these working memory tasks were contrasted against control tasks (e.g., 0‐back) or rest periods. Hippocampal sources estimated either from evoked potentials or from oscillatory activity (theta and slow‐gamma bands) were often found to be more active in the memory‐demanding tasks, but the reported temporal effects lasted no longer than 50 ms. However, the analyses generally included data from the whole tasks which comprised “repeated” or “new” stimuli (Luckhoo et al., 2012; Quraan et al., 2011); only one study separated trial types (Hung et al., 2013) and found hippocampal activations due to higher working memory load not only during successful recognition but also during the encoding of novel items.

There is an additional published paper (Staudigl & Hanslmayr, 2013) of relevance here that fulfills our conceptual selection criteria but was not detected in our search because it does not have the term “MEG” in the title, abstract, or other PubMed‐indexed fields. Their study reports on subsequent memory effects (i.e., neural responses when encoding later remembered information or “hits”, relative to that of forgotten information or “misses”) with opposite signs, depending on whether or not the background context of to‐be‐memorized words was presented again in the retrieval phase. Some of these interaction effects were expressed as modulations in theta power (3.5–4.5 Hz) and theta‐to‐gamma phase‐amplitude coupling in left hippocampal sources, demonstrating the suspected link between these oscillatory correlates and item‐context binding. As with the studies reviewed above, the authors designed a hippocampal‐dependent task, computed realistic single‐shell brain models for the healthy participants, and applied a beamformer variant (DICS). Detection of hippocampal sources may have been further facilitated because, for each subject, the “miss” activation map was subtracted from the “hit” activation map before applying the contrast between context conditions; thus, the “miss” acted as a control condition.

The remaining studies employed spatial navigation tasks based on the Morris water maze paradigm, originally used in animal studies and adapted for humans by means of computer simulations. In these tasks, participants are asked to learn the location of items within a virtual horizontal plane surrounded by distal landmarks. To be able to remember the correct location at test, participants must develop an allocentric cognitive map from that distal information, a faculty for which hippocampus is critical. All studies localized hippocampal sources after correlations were tested between regional oscillatory power and different measures of spatial performance (Cornwell et al., 2012; Cornwell et al., 2014; Cornwell, Johnson, et al., 2008; Crespo‐García et al., 2016; Kaplan et al., 2012; Kaplan et al., 2014). Most of these studies found positive relationships between 4–8 Hz hippocampal theta during goal‐directed navigation and subsequent performance, consistent with the well‐known link between theta rhythm and movement (Whishaw and Vanderwolf, 1973). In contrast, Crespo‐García et al. (2016) combined the navigation paradigm with a classical subsequent memory task and investigated theta activity, not only during active navigation but also during the encoding of picture‐location associations. They found negative correlations between a slower (2–3 Hz) hippocampal band and spatial memory accuracy, effects observed both in MEG from healthy subjects and iEEG collected from a group of patients. These opposite correlation patterns might reflect the existence of two hippocampal theta rhythms with dissociable roles in memory and locomotion (Lega et al., 2012). Evidence that theta power decreases benefit episodic memory formation has been gathered with iEEG (e.g., Long et al., 2014; Greenberg, Burke, Haque, Kahana, & Zaghloul, 2015) and with surface EEG in real‐world spatial contexts (Griffiths et al., 2016). Furthermore, in a single‐case analysis of interictal MEG part of the same study, Crespo‐García et al. found that hippocampal sources showing slow‐theta decreases established phase interactions with the left temporal cortex, a result that was validated using either simultaneously recorded iEEG cortical signals or equivalent beamforming sources.

#### 3.3.3 Perception studies

A minority of studies retrieved by this review had a particular interest in hippocampal function in relation to perceptual processes, which may arguably be distinct from memory encoding processes. For instance, Kveraga et al. (2011) focused on using MEG in combination with fMRI to identify neural networks that are activated as a part of the top‐down context evaluation that already occurs during early object recognition. Turning from the visual to the auditory, Barascud et al. (2016) found similar networks that are activated when participants are asked to identify patterns in acoustic sequences, while Fujioka, Zendel, and Ross (2010) found the hippocampus to be involved in a distributed network used by musicians for temporal processing during timing detection.

#### 3.3.4 Decision making studies

Finally, one study by Guitart‐Masip et al. (2013) focused on the role of the hippocampus in decision making, finding that MTL sources generated increased theta activity during a nonspatial decision‐making task. Such activity is attributed to the anterior hippocampus because of its close proximity to the MTL and that motivational and emotional behavior — including single item (noncontextual) memory — are attributed to the structure (Fanselow & Dong, 2010). While the paradigm was meant to identify differences in activations in relation to the expected value of choices, the analysis resulted in a lack of contrast for value. As such, the authors attribute a correlation in theta power between anterior hippocampal and prefrontal sources with a mnemonic process of human decision making.

### 3.4 Clinical studies: A detailed evaluation

In addition to those studies that investigated normal hippocampal function, we also found 12 patient studies that can be broken into the following clinical diagnostic categories: studies of patients with hippocampal damage and epilepsy, those with schizophrenia, those with depression and anxiety, those with concussion, and those with PTSD. Broadly, we noted that studies that included patient groups more commonly (i.e., 7 of 12, see Table 1) evaluated resting state MEG recordings (perhaps because they are easier to acquire in these populations) and thus were more likely to focus on the difference between resting state parahippocampal network oscillatory activity for cases and controls; however, some simple functional tasks were also used.

#### 3.4.1 Hippocampal damage and epilepsy

There is in fact a sizeable body of research that uses MEG to investigate hippocampal damage and epilepsy, but the majority of these clinical studies were excluded from this review due to the presence of epileptic spike activity or the use of single dipole source reconstruction methods. Crespo‐García et al. (2016), meanwhile, used patients only to validate neurotypical results and is therefore discussed in section 3.2 and 3.3 above. One key paper relating to hippocampal damage remains (Chatani et al., 2016). In this study, patients with mesial temporal lobe epilepsy and unilateral hippocampal sclerosis were contrasted with healthy individuals and patient controls to demonstrate that auditory‐evoked magnetic fields are influenced by hippocampal inputs. It should be noted, however, that this work lacks MEG‐based localization of hippocampal sources but rather implicates hippocampal damage in the reduction of MEG‐detected auditory source activity.

#### 3.4.2 Schizophrenia

Individuals with schizophrenia are of particular interest to the hippocampal research community due to the memory impairments characteristic of this disorder. Previous neuroimaging research has furthermore uncovered structural and functional differences (Fornara, Papagno, & Berlingeri, 2017; Ota et al., 2017; Pirnia et al., 2015; Ragland et al., 2015; Seidman et al., 2014) linked to hippocampus in schizophrenia. Here, MEG can be used to determine what temporal differences in neural processing might exist for those with this psychiatric condition. A paper by Hanlon et al. (2011), for instance, successfully used the transverse patterning task previously used in healthy individuals to evaluate hippocampal activity during verbal and nonverbal tasks. Unusually, this study used sLORETA (a weighted L2 minimum‐norm approach for source localization) and found an atypical lateralization pattern for individuals with schizophrenia as compared to controls. Other studies have used beamformers to localize hippocampal activity in patients with schizophrenia: in a study of auditory verbal hallucinations, van Lutterveld et al. (2012) found a decrease in right hippocampal theta power with a spatial filter SAM method, while Cousijn et al. (2015) used an LCMV beamformer to identify a decrease in intra‐hippocampal theta in healthy individuals with an elevated genetic risk for schizophrenia as compared to neurotypical controls.

#### 3.4.3 Depression and anxiety

A number of studies included in this review focused on depression, major depressive disorder (MDD), and anxiety. One such study, Cornwell et al. (2010) used a virtual water maze similar to the one described in other papers by the authors, discussed in section 3.3.2 (Cornwell et al., 2012; Cornwell et al., 2014; Cornwell, Johnson, et al., 2008). Here, it was found that patients had impaired spatial navigation and differences in bilateral parahippocampal theta activity. Specifically, left posterior hippocampal theta was found to be correlated with behavioral performance: patients, in general, demonstrated less activity in the anterior hippocampus and parahippocampal cortices as compared to controls. In addition, a resting state study of depression included in this review used SAM beamforming source analysis and the ICA method to describe resting state networks in individuals with MDD and found reduced correlations in networks linked to the hippocampus (Nugent, Robinson, Coppola, Furey, & Zarate, 2015). We also found one study examining the role of the hippocampus in healthy individuals performing a task designed to induce anxiety: oscillatory power, particularly in gamma band, was linked to threat probability (Khemka, Barnes, Dolan, & Bach, 2017).

#### 3.4.4 Concussion

One study examined the effects of mild concussion on a variety of cortical and subcortical structures associated with memory and attention. Using a simple working memory task, this study found a range of atypical hypo‐ and hyper‐activation patterns in individuals with concussion, even where no behavioral differences were apparent (Shah‐Basak et al., 2017). Here, for the concussion patients, right hippocampus exhibited greater activation.

#### 3.4.5 Posttraumatic stress disorder

PTSD is a disorder that intimately involves personal memories and experiences, and makes up the final category of clinical studies using MEG to investigate hippocampal function. We found four studies that measured hippocampal function; in every case, resting state recordings were used. One challenge for these studies was to find an appropriate control group. Multiple groups were used as a contrast relative to the main group of interest, ranging from healthy civilians to non‐PTSD individuals on active combat duty. Overall, these studies found a wide variety of differences in hippocampal and parahippocampal activity during resting state in individuals with PTSD. This included increased activity in several brain regions including hippocampus (Badura‐Brack et al., 2017) and in beta, gamma, and high‐gamma frequency bands, while other areas had decreased activity in lower‐frequency bands (Huang et al., 2014). Long‐range hyperconnectivity (regarding the control group) in these same high‐frequency bands involving the left hippocampus, temporal, and frontal regions was also reported and, in particular, correlations with scores on the PTSD Checklist and left hippocampal activity were found (Dunkley et al., 2014). Furthermore, these left‐hemisphere and high‐frequency differences also correlated to a reduced dynamic range of neural activity, as measured by local signal variability (Mišić et al., 2016). It is speculated that robust differences in temporal signaling could be used as a biomarker for the condition.

### 3.5 Hippocampal dynamics detected with MEG

Several empirical studies analyzed hippocampal activations relative to events or participants' responses. Most of them detected significant experimental modulations of these activations that lasted a few hundred ms. Whether or not these effects truly express the temporal dynamics of hippocampal engagement is difficult to assure without ground truth data. However, some of the signals agree with expected hippocampal activity patterns derived from observations with animal model and invasive studies on humans. For example, Kaplan et al. (2012) found an increase in theta power in the right hippocampus shortly after the initiation of voluntary movements during virtual navigation relative to stationary periods. Previously, Cornwell et al. (2008,b) had reported a similar effect in the left hippocampus when comparing goal‐directed to aimless movements in a virtual pool. These effects replicate well‐known demonstrations of movement‐related theta activity in the hippocampus of rats (Vanderwolf, 1969) and humans (Ekstrom et al., 2005). Human invasive studies (Greenberg et al., 2015; Lega et al., 2012) have also validated poststimulus subsequent memory effects in the theta band, including time intervals like those reported here (Backus et al., 2016; Crespo‐García et al., 2016; Kaplan et al., 2012). A decision‐making study (Guitart‐Masip et al., 2013) found increased phase synchronization between the hippocampus and prefrontal cortex in the theta band, as seen in rodents during spatial memory tasks (Jones & Wilson, 2005). An equivalent MEG correlate was also found during cue periods where participants presumably retrieved the spatial location of objects (Kaplan et al., 2014); this connects with another virtual navigation study with human intracranial recordings showing increased connectivity between parahippocampus and lateral prefrontal cortex during spatial context retrieval (Watrous et al., 2013). Finally, in the auditory modality, MEG responses in the hippocampus were found to be distinguishable at different latencies depending on the meter and accent of the stimulus (Fujioka et al., 2010), or whether complex sound sequences are perceived to be regular or random (Barascud et al., 2016). Accordingly, auditory evoked responses in the human hippocampus have been previously demonstrated with iEEG, where peak latencies were found to be sensitive to whether the stimulus was a target or distractor in an oddball task (Halgren et al., 1995).

## 4 DISCUSSION

In this review, we set out to examine whether MEG methods can be used to effectively localize hippocampal activity. We found that MEG, combined with adequate methodological paradigms, can be usefully employed for sensing activity originating in the hippocampus and parahippocampal networks. Furthermore, the evidence shows that it is in fact possible not only to discriminate hippocampal signals from the cortical background noise but also to reliably localize these signals to hippocampal structures. It is worth mentioning the possibility that MEG‐detected activations that are attributed to the hippocampus are instead generated by other sources in close proximity to it. An explicit example of this possible ambiguity is described in section 3.3.4: Guitart‐Masip et al. (2013) localize signals to the MTL but attribute them to the hippocampus. However, while that work focused on decision‐making (perhaps underlying their conservative presentation of possible sources), the evidence for memory and spatial‐navigation related functional activations being associated with the hippocampus (as opposed to structures close by, including MTL) is much stronger. (Because the hippocampus is activated by a broad range of tasks and functions, it seems likely that hippocampal activations are falsely mapped to cortical regions in proximity to it as a result of the fact that standard MEG analysis packages lack hippocampal sources).

The studies found in our search generally confirm or expand on results from experiments with both animal models and humans. What is unique is timing: MEG can, and has, been used to reveal top‐down versus bottom‐up processing and elucidate the dynamics of memory retrieval (e.g., relative to tasks/stimuli, c.f., section 3.5). However, the historical lack of consensus regarding whether MEG is sensitive to the hippocampus as well as the challenges associated with properly identifying hippocampal signaling may have tempered the depth of interpretation taken with respect to timing and dynamics. This, however, seems to be changing as many of the more recent papers (e.g., Backus et al., 2016; Crespo‐García et al., 2016; Garrido et al., 2015 and Kaplan et al., 2017) place emphasis on dynamics (rather than localization in and of itself). If confidence in localization and estimation of dynamics continues to improve, one can expect the study of hippocampal connectivity and network function to grow. The reliability and feasibility of MEG hippocampus studies can be further advanced via the following.

### 4.1 Key findings and recommendations

A synthesis of the papers reviewed indicates that, beyond the general recommendations for a successful MEG experiment (c.f., Hari & Salmelin, 2012; Gross et al., 2013; Hari et al., 2018), there are two main parts of a MEG‐based study of hippocampal function that are worthy of careful consideration which we list here and explain in more detail below:

#### 4.1.1 Experimental design

Paradigms should be designed such that:

- hippocampal contrast can be maximized, for example, by having at least two types of trials that are expected to induce the same activations in all brain areas *except* the hippocampus.
- the timing of expected hippocampal activations can be reliably annotated, for example, using tasks that force recall to occur within an experimentally controlled time window.
- mnemonic states can be grouped (and thus averaged), for example, successful versus unsuccessful trails can be used for independently contrasting recall, encoding, and/or spatial navigation.
- the co‐registration of the head position in the MEG session with the individual's MRI is performed with high precision and accuracy. This can be done via digitization of the head surface and fiducials combined with the use of head position indicator coils that allow continuous monitoring of the head position (Uutela, Taulu, & Hämäläinen, 2001) or head casts that fix the head position during a MEG session (Meyer et al., 2017).

#### 4.1.2 Analysis methods

Data processing pipelines should:

- include physiologically constrained models of the hippocampus in the forward model and thus inverse operator.
- use distributed source (e.g., with minimum‐norm) or scanning‐based (e.g., with beamformers) inverse methods instead of equivalent current dipoles.
- include beamformer‐based source estimates to improve comparability to the existing literature (29 of 37 of the papers reviewed relied on beamformers, but this may be a result of the historical reliance on beamformers for analysis of oscillatory neural activity and should not be taken as evidence that minimum‐norm or other distributed source modeling methods are inappropriate for estimating hippocampal activations).

A key to successful localization is thoughtful experimental design. First, a good theoretical as well as physiological basis for the experimental procedures can help to ground results in a specific hypothesis. For instance, the use of spatial tasks such as the water maze is supported by equivalent experiments in rodents and established research strongly supports that spatial learning is hippocampal‐dependent and engages associative and path integration networks that are functionally connected with the hippocampus (see Vorhees & Williams, 2014 for a review). Furthermore, the use of established memory and spatial navigation tasks may have more success in activating hippocampus than resting state or other less theoretically relevant tasks. (In fact, resting state recordings could potentially elicit hippocampal activations because subjects may spontaneously remember past episodes as their minds wander, but this activity is not controlled by the researcher or the research paradigm and is difficult to annotate.)

This review uncovered an additional point of value, consistent with e.g., fMRI research (Simó et al., 2015): although hippocampal activations can be detected during any recognition task, effects are likely to be most pronounced during the encoding of novel information, that is, during novel and relational encoding paradigms. This also meshes with our finding of several studies demonstrating hippocampal activation during early stages of perception, perhaps indicative of top‐down processing associated with placing a stimulus in context or evaluating it in some way. To summarize, our results indicate that MEG studies are more likely to detect hippocampal sources when investigating memory encoding of novel relationships, or flexible spatial learning (Table 1).

The importance of developing refined experimental paradigms is a crucial point in identifying signals from the hippocampus. When planning an experiment, it is valuable to consider how best to isolate hippocampal activity, by avoiding design elements that may create a strong visual or sensorimotor cortical response, by desynchronizing hippocampal activity from other activity, and/or by providing clear control conditions as contrast. MEG research, along with EEG and fMRI studies, relies heavily on improving SNRs by repeating a stimulus many times and then averaging across trials. Even for analysis of resting state data, analysis methods frequently resort to dividing continuous data into short chunks and performing analogous averaging. A conundrum thus arises: while trial averaging would facilitate the detection of hippocampal signals, from a conceptual perspective memory studies are not easily adapted to commonly used stimulus‐repetition experimental designs. However, memory paradigms do allow grouping trials that are assumed to be processed under a similar mnemonic state: for example, in a subsequent memory task, all stimuli that during the encoding phase were posteriorly remembered or forgotten; or during the retrieval phase were successfully or unsuccessfully recognized, and so forth This strategy enables the study of an averaged correlate of some memory condition that, in light of our review, can boost the detection of hippocampal sources as well.

Beyond this, it is recommended to use tasks that will allow for sufficient trials per condition and participants per group to increase SNR. The trend toward inclusion of more subjects (c.f., Table 1) suggests that the burden of proof has risen over the past decade, but (perhaps more importantly) there is a general willingness in the field to commit more resources to MEG studies of the hippocampus. Ideal tasks will be those known to induce hippocampal activity, and will also employ a controlled contrast that does not recruit hippocampus (e.g., a visual task that does not employ memory).

Finally, it is worth considering even at a planning stage how the resultant data will be processed — how to reduce leakage with spatial filters, how to apply beamformers to contrasting conditions, and so on. Currently, beamforming methods appear to be most popular with research groups attempting to localize hippocampal activity, although this may be in part due to the focus on oscillatory observations rather than evoked activity.

Beyond this, the importance of forward models should be considered: in the past, boundary element models (BEM) were extremely time‐intensive to create, but more automated MRI segmentation strategies, increased processing power, and more accessible software have led to greater speed and ease of use in creating them today. Yet, somewhat surprisingly, many of the included studies used forward models based on template MRIs rather than individual anatomy. This is, in fact, encouraging for future research because it implies that, at least for group studies, it may often be achievable to discern hippocampus effects with MEG in the absence of an accompanying structural MRI (Douw, Nieboer, Stam, Tewarie, & Hillebrand, 2018; Henson et al., 2009; Holliday, Barnes, Hillebrand, & Singh, 2003).

Future advances will doubtless provide additional improvements and lead to more confidence in source space modeling. From this review, an interesting approach was leveraged by Backus et al. (2016), who used a lead field orthogonalization method to help minimize the impact of leakage from other regions on the hippocampus source reconstruction. Continued development of source localization and analysis methods may allow pinpointing of transient signals during memory encoding and other hippocampal tasks.

Interestingly, one final observation from this review is the divide between clinical and nonclinical studies. While some researchers do attempt to translate findings from healthy participants to disease models (Cornwell et al., 2008,b, 2010, 2012, 2014), much neuropsychiatric research remains exclusively informed by lesion studies (Szczepanski & Knight, 2014) rather than the body of animal research and behavioral psychology experiments. The prevalence of resting state paradigms in clinical studies, for which analyses are generally limited to network connectivity, precludes within‐subject imaging contrast that strengthens the case for hippocampal sensitivity. Albeit that such investigations involving neurological, developmental, and mood disorder conditions are often constrained by practical considerations including the need for simple tasks, the value of utilizing well‐developed paradigms that target imaging contrast in the hippocampus cannot be understated. As with the Morris water maze, paradigms developed for hippocampal studies in animal models can inform research experiments that are likely to be tolerable for a broad range of clinical presentations. Other practical limitations including the heterogeneity and relatively small sample sizes of these clinical participant groups can then be partially alleviated with higher SNRs and imaging contrast for more definitive functional localization. With these considerations in mind, we believe that the power of hippocampus‐based physiological biomarkers in clinical studies is then likely to improve in the future.

### 4.2 Future directions

The findings of this review are accompanied by a range of outstanding questions that remain to be addressed as well as recommendations for future studies. These vary from the methodological to the theoretical.

While undertaking the review process, we hoped to find alternative methods that would clearly corroborate (or refute) MEG localization of hippocampal activity. Ultimately, only a minority of our resultant papers accompanied MEG with other measures, and (though Mills et al., 2012 does use contrast analysis techniques) none had a specific aim to directly contrast imaging methods. Given that fMRI is the most prevalent noninvasive technique for assessing hippocampal function, and iEEG is the only way to obtain a direct electrophysiological reading of the working human brain, we express the hope that more work is done to align these diverse methods into a united consensus and to highlight their complementary properties.

We would like to briefly note here several iEEG/ECoG studies that, while excluded from this review due to the presence of epileptiform activity or lack of modern localization methods, may still provide additional insight for future researchers – iEEG findings may support and validate MEG source localization methods, as well as provide some direction for future improvement strategies (Dalal et al., 2013). First, we acknowledge the study by Knowlton et al. (1997), briefly mentioned in explaining our paper selection criteria, which focused on the localization of spike activity in epilepsy and the measurement of this activity by MEG in contrast to EEG and fMRI, and reports that MEG can reliably localize epileptiform activity and can sometimes provide additional data for clinical patients. Again, it is worth reiterating that SNR is generally higher for epileptiform spikes, simplifying the localization process. Further, while studies do regularly indicate that the inclusion of MEG data may confirm or improve the identification of a seizure focus (Assaf et al., 2004; Smith et al., 2000; Stefan et al., 1991; Stefan et al., 1994), the process is not perfect and erroneous localizations can still occur: a pair of simultaneous iEEG and MEG studies (Hisada, Morioka, Nishio, Yamamoto, & Fukui, 2001; Shigeto et al., 2002), for instance, demonstrate that even when SNR is high, there may still be a mismatch between identified seizure onset zone when using equivalent current dipole‐based localization.

In addition to our recommendation for future studies exploring the differences between these various imaging modalities, we also would like to note the expanding field of MEG and simulation or modeling data (Attal & Schwartz, 2013; Balderston, Schultz, Baillet, & Helmstetter, 2013; Mills et al., 2012; Quraan et al., 2011; Stephen, Ranken, Aine, Weisend, & Shih, 2005). Currently, studies such as the recently published Meyer et al., 2017,b continue to elegantly demonstrate the theoretical ability of MEG to robustly detect hippocampal activity. However, the field would benefit from more work done to unite findings from simulation data with experimental data. For example, the spatial extent of source estimates from experimental data could be compared to theoretical analyses of the point spread/contrast transfer functions for hippocampal sources. Purely theoretical analyses aimed at improving the understanding of potential confounds and characterizing the limitations of localizing activity to the hippocampus are furthermore critical to the field.

Improved confidence in the localization of MEG‐detected hippocampal activations would benefit from a more detailed anatomical model of the hippocampus, for example, via high‐resolution 7 T MR‐imaging. Simulations with such a model can furthermore provide powerful insight regarding the specific hippocampal regions to which MEG can and cannot be sensitive. The inclusion of such models in commonly used MEG analysis packages would benefit not only those studying the hippocampus but perhaps also the more general MEG community as well.

One outstanding question that MEG may have the potential to answer (but which has not yet been fully clarified) is the extent to which the hippocampus is involved in working memory as compared to long‐term memory, and further the precise parahippocampal sequence of activation for memory encoding and retrieval in humans. Because of the limitations of fMRI and animal studies, there is still some ambiguity regarding what aspects of memory actively recruit the hippocampus, and what segments of the hippocampus are differentially involved in memory construction and reconstruction. When combined with carefully constructed behavioral tasks, the fine temporal resolution of MEG may help to elucidate these processes in humans.

A promising methodological approach to investigate transient brain activations is the application of multivariate pattern classification (MVPC) to neural oscillations recorded with MEG. Among the reviewed studies, one of them used this strategy to identify beta and gamma activity patterns that were signatures of mnemonic reactivations during the maintenance period of a delay match‐to‐sample task (Poch et al., 2011). By computing the phase locking of these reactivations to theta oscillations and associating the output with memory performance, hippocampal sources were highlighted. A recent study applied a MVPC approach at MEG signals recorded during a task where participants selected nonspatial paths between visual objects to get a monetary reward (Kurth‐Nelson, Economides, Dolan, & Dayan, 2016). The pattern classifiers were trained on activity measured during the presentation of single objects and were tested during the planning period when no objects were presented. The experimenters were able to decode 120‐ms sequences of about four objects, replayed in a reverse order with respect to the transitions made during the task. Although the MVPC was trained on sensor‐level data, it could potentially be combined with source localization and help to decode hippocampal mnemonic representations as well. Indeed, there is compelling evidence for temporal order memory encoded by theta‐gamma coupling in hippocampal sources (Heusser, Poeppel, Ezzyat, & Davachi, 2016), which strongly support our thesis that accurate temporal information from hippocampus can be assessed with MEG when using an appropriate methodology.

A MEG study (Stephen et al., 2005) using simulated interictal activity at different subfields of the hippocampus, parahippocampal cortex, and temporal cortex, shed some hope regarding the spatiotemporal resolution of signals generated at these structures. Results showed that although hippocampal sources from different subfields were not resolvable, the location and orientation of neocortical sources was differentiable from MTL sources, and hippocampal sources were distinguishable from parahippocampal sources except when the waveforms overlapped in time. The ability to differentiate hippocampal from neocortical sources offers an additional advantage when investigating large‐scale hippocampal dynamics with MEG. Although we could not obtain an estimate of the spatiotemporal accuracy of these dynamics from the empirical papers, we repeatedly found patterns of theta phase coupling between hippocampus and prefrontal cortex across different studies evaluating decision making (Guitart‐Masip et al., 2013), memory retrieval of spatial locations (Kaplan et al., 2014), short‐term memory maintenance (Poch et al., 2011), and memory integration (Backus et al., 2016), consistent with animal models. Nevertheless, there are still methodological limitations like volume conduction and leakage that could reduce the spatial resolution of hippocampal MEG activations; this aspect may be critical to disambiguate hippocampal effects from those of surrounding sources, or when investigating different roles of anterior and posterior hippocampus.

While a majority of studies use naturalistic tasks that have clear animal correlates (e.g., the Morris water maze), other, more abstract, paradigms such as the transverse patterning task may provide additional fine‐grained resolution for describing hippocampal function. This task in particular may provide an opportunity to study activity during initial learning and encoding phases, and has previously allowed researchers to demonstrate that simply changing the type of stimuli used can cause hippocampal activity to increase in strength (Moses et al., 2009) or lateralize to one hemisphere (Hanlon et al., 2011). In the future, additional deconstruction and fine‐tuning of experimental paradigms may lead to a better understanding of the time‐course of hippocampal signaling.

It remains an open question as to whether MEG recordings of activity can be localized to the hippocampus with a level of confidence that will allow future researchers to include the structure as a potential source in paradigms that are not hippocampus‐specific; currently, this is presumably beyond the reach of state‐of‐the‐art systems. We encourage the execution of a meta‐analysis via a quantitative review of the data presented in the works cited in Table 1. Such an effort will require collaboration with as many of the groups that perform MEG recordings of hippocampal function as possible as the results are presently not directly comparable with a statistical approach.

Advances in MEG sensor technology, such as the potential for improved spatial resolution via MEG with high‐Tc SQUIDs or optically pumped magnetometers, provide a tantalizing glimpse of what may be in store for the future. The relaxed thermal insulation requirements of newer magnetic sensor technologies compared to conventional SQUIDs, including high critical temperature SQUIDs (Andersen et al., 2017; Riaz, Pfeiffer, & Schneiderman, 2017) and optically‐pumped magnetometers (Boto et al., 2017; Iivanainen, Stenroos, & Parkkonen, 2017), enable on‐scalp MEG wherein improved proximity to the hippocampus can lead to higher signal levels. High‐Tc SQUID‐based MEG, which takes advantage of advancements in superconducting sensor technology, aims to use liquid nitrogen cooling systems in lieu of liquid helium, while optical magnetometers operate near room temperature. These systems, still in development, are demonstrated to have comparable or better SNRs to classic MEG, and suggest their potential utility to measure a broader range of brain activity from deeper structures (Boto et al., 2016; Boto et al., 2018; Iivanainen et al., 2017; Öisjöen et al., 2012; Schneiderman, 2014). However, given the importance of the hippocampus as a hub for a variety of higher cognitive functions, no doubt any advances will be instrumental not just for our understanding of this one deep structure, but for our understanding of human development and behavior as a whole.

### 4.3 Limitations of the current review

As with all review studies, a number of limitations exist that may mitigate the current findings. Despite our best efforts to use prespecified criteria to minimize bias, we may have missed valuable sources, for instance through our search strategy being confined to a database like PubMed (which has not implemented full‐text search and may miss relevant literature), or through the rigor of our inclusion and exclusion criteria. Given their relevance, two additional studies missed in the search were included above (Heusser et al., 2016; Staudigl & Hanslmayr, 2013); but it is not unlikely that there are more.

In addition, this review may suffer from a problem that reaches beyond the current subject, namely publication bias and the bottleneck that prevents scientific findings, in particular, null findings, from being seen by the broader scientific community. It has been observed that many peer‐reviewed publications preferentially publish novel studies that refute the null hypothesis, so it is likely that our review is missing studies that attempted and failed to localize hippocampal activity or studies that simply replicated already‐published findings.

More specifically, we aimed to overcome methodological and epistemological heterogeneity through our qualitative method of review, but it should be kept in mind that the studies included in this review have a diversity of focus, not to mention differences in study design. For instance, papers that focus on methodological concerns may have relatively little that can be coherently synthesized with those that have a clinical focus. This seemingly unavoidable limitation is likely due to the heterogeneity of hippocampus research, which in turn arises from the multitude of functions ascribed to this deep structure.

Finally, we selected inclusion and exclusion criteria that use clear constraints that would be most likely to provide interpretable and synthesizable data. For example, we excluded studies that used older methodologies such as equivalent dipole methods because we, along with the community at large today view these as insufficiently sensitive for localizing sources with low SNRs, whose components are longer, later, or more variable, or that are easily masked by more dominant shallow sources. However, it is inevitable that our criteria excluded some papers that may have provided additional insights.

## 5 CONCLUSIONS

In sum, we find that continued developments in the field of MEG research are increasingly making it possible to use this method to detect electrophysiological activity that is understood to be generated by the hippocampus. Advanced methods and improved models, established in conjunction with other discoveries from iEEG, fMRI, and other neuroimaging arenas, have been key to this success. Combining these methods can result in findings that are more than the sum of their parts (Cornwell, Carver, et al., 2008; Hall, Robson, Morris, & Brookes, 2014; Hipp & Siegel, 2015; Schulz et al., 2004; Singh, Barnes, Hillebrand, Forde, & Williams, 2002) and will likely improve our understanding of cognition and brain activity (Freeman, Ahlfors, & Menon, 2009; Huster, Debener, Eichele, & Herrmann, 2012; Liu, Ding, & He, 2006; Mullinger & Bowtell, 2010) and lead to greater insights in timing and network modeling (Hari & Salmelin, 2012).

Key takeaways for future research can be summarized as follows: (a) we recommend considered planning in the development stage of a study, prioritizing standard paradigms (i.e., memory and navigation tasks that tap into encoding and retrieval mechanisms) for selectively activating hippocampus through the use of experimental tasks already established through animal studies or otherwise grounded in theoretical understanding of hippocampal function; (b) following on from this point, we suggest the use of a contrast‐based experimental design to mitigate the influence of, for example, dominating sensory activations; (c) as with all neuroimaging studies, but especially for investigations of hard‐to‐localize structures, it is crucial to gather substantial data to improve SNR, by employing a sufficient number of trials from a sufficient number of participants; (d) finally, we identify the challenge of selecting adequate modeling methods for integrating a physiologically relevant reconstruction of the hippocampus with standard MEG analysis source models. In addition to these recommendations, we encourage continued critical investigations that attempt to compare and contrast various theoretical and/or electrophysiological reconstruction techniques for MEG, as well as complementary methods (including fMRI and iEEG).

While challenges and questions still remain, the detection of hippocampal activity with MEG has made significant strides in recent years, and the next generation of MEG sensor technology, together with more accurate forward models and clever source localization strategies, may yield yet further gains. These developments will strengthen our arsenal of tools for investigation the human hippocampus noninvasively and lead to a greater understanding of its dynamics.

## CONFLICT OF INTEREST

The authors have no conflict of interest, financial or otherwise, related to this work.
